# Baclofen intoxication after accidental ingestion in a 3-year-old child

**DOI:** 10.4103/0253-7613.51349

**Published:** 2009-04

**Authors:** Nagesh Dasarwar, Preeti Shanbag, Nilesh Kumbhare

**Affiliations:** Pediatric Intensive Care Unit, Department of Pediatrics, Lokmanya Tilak Municipal Medical College and General Hospital, Sion, Mumbai - 400 022, India

**Keywords:** Accidental ingestion, baclofen, toxicity

## Abstract

Baclofen is a skeletal muscle relaxant, used to control spasticity in both adults and children with neuromuscular disorders. Several cases of baclofen overdose have been reported, but only a small number have involved children. We report a 3-year-old girl with accidental ingestion of baclofen, who presented with coma, bradycardia and hypotension. She recovered within 24 hours with supportive treatment. The case emphasizes the importance of warning parents about the potential toxicity of baclofen when the drug is prescribed to a family member.

## Introduction

Baclofen is an analog of the naturally-occurring inhibitory neurotransmitter gamma-aminobutyric acid (GABA) found in the central nervous system. Specifically, baclofen binds to GABA β receptors, inhibiting the influx of calcium and thus preventing the release of the excitatory neurotransmitters glutamate and aspartate.[1]

The drug is administered orally to treat spasticity associated with spinal lesions such as traumatic injuries and demyelination as in multiple sclerosis. It is used intrathecally in treating spasticity associated with supraspinal lesions like cerebral palsy and stroke.[1] Baclofen has also been used for treating chronic hiccups and for cocaine abuse.[2,3] Lately, it has also become popular as a recreational drug.[4] Baclofen overdose can result in coma, seizures, respiratory depression, cardiac rhythm abnormalities and rhabdomyolysis.[4,5]

We describe a case of baclofen toxicity presenting with coma, flaccidity, hyporeflexia, bradycardia and hypotension.

## Case Report

A previously well 3-year-old girl presented to us with sudden onset of unresponsiveness. She was brought to the hospital within half an hour of onset of symptoms. There was no history of fever, convulsions or head injury. Despite inquiry no history of drug ingestion could be elicited. The child's development was appropriate for her age. Physical examination revealed a deeply comatose child with a Glasgow Coma Scale (GCS) score of 3. She was normothermic with a heart rate of 70/minute, respiratory rate of 24/minute and blood pressure of 62/40 mmHg. There was no icterus or pallor, nor were there any signs of injury. There was no peculiar odor to the breath or the clothes. The weight was 13 kg (25^th^ percentile for age). The pupils were neither pinpoint nor were they dilated, and were reacting well to light. Extra-ocular movements were normal. Generalized hypotonia was present and the deep tendon reflexes could not be elicited. There was no focal neurological deficit, nor signs of meningeal irritation. The fundus showed no papilledema or hemorrhages. On examination of the respiratory system, breath sounds were normally heard and there were no adventitious sounds. Examination of the abdomen revealed no hepato-splenomegaly.

In view of the low blood pressure, the child was given an intravenous bolus of 20 ml/kg of normal saline and continued on intravenous fluids and dopamine. Intravenous ceftriaxone was started for suspected meningo-encephalitis and discontinued after 48 hours when the CSF culture was reported as sterile.

Blood sugar at admission was 108 mg/dL. Pulse oximetry revealed an oxygen saturation of 98%. The hemoglobin was 10.3 g/dL. The total WBC count was 17,800/mm^3^ with a differential of lymphocytes 64%, polymorphs 34% and monocytes 2% and the platelet count 3,47,000/mm^3^. C-reactive protein was 5.6 mg/L (cut-off for bacterial infection in children 35-60 mg/L). Lumbar puncture done after stabilization of the child showed 2 cells/mm^3^, both lymphocytes. Cerebrospinal spinal fluid (CSF) sugar was 64 mg/dL and protein 42 mg/dL. CSF culture was reported as sterile. A computed tomography (CT) scan of the brain was normal. The blood urea nitrogen was 3 mg/dL, serum creatinine was 0.9 mg/dL. The liver function tests showed a total protein of 6.9 g/dL, albumin 4 g/dL, globulin 2.9 g/dL and total serum bilirubin 0.9 mg/dL. Serum aspartate aminotransferase (AST) was 19 U/L, serum alanine aminotransferase (ALT) 33 U/L and serum alkaline phosphatase 277 U/L. The electrocardiogram showed sinus bradycardia.

Six hours after admission, additional history given by an older sibling revealed that the child had been playing with some tablets and had swallowed some. The father had been prescribed baclofen (Lioresal) for frequent hiccups and on checking found six 10 mg tablets of baclofen missing. The exact time of ingestion of the tablets could not be ascertained. Gastric lavage was subsequently done. Since baclofen is excreted primarily via the kidney, the patient was started on vigorous hydration and forced alkaline diuresis. Five hours after starting this therapy, the child showed signs of improvement in the form of an increase in the heart rate to 92/minute, stable blood pressure and increase in the Glasgow Coma Scale to 10 (E3M4V3). Within 24 hours the child had regained normal consciousness, and heart rate and blood pressure were normal. The child was transferred to the general ward after 48 hours and subsequently discharged.

## Discussion

Our patient presented with coma, hypotonia, hyporeflexia, bradycardia and hypotension.

Symptoms of baclofen toxicity generally appear within 2 to 6 hours of ingestion and include hypoventilation, coma, flaccidity, hyporeflexia and hypothermia. Autonomic disturbances are frequent but inconsistent; patients may have either bradycardia or tachycardia, hypotension or hypertension, and miosis or mydriasis. Seizures and conduction abnormalities are also seen.[6,7] Seizures are generally brief, tonic-clonic, and respond rapidly to pharmacological intervention. Cardiac abnormalities observed include supraventricular tachycardia, premature atrial contractions, first degree heart block and prolonged QTc.[4,7] Hallucinations may also occur, but are typically seen in patients who are chronically medicated with baclofen, who receive an acute overdose.[8] Rhabdomyolysis has also been described.[5] Laboratory abnormalities have included transient elevations of lactic dehydrogenase, AST, alkaline phosphatase and blood glucose.[4]

The management of baclofen toxicity is mainly supportive.[4] Gastric lavage and activated charcoal administration has been recommended for children who ingest more than 5 mg/kg. The patient should be stabilized by maintaining the airway, breathing and circulation. Intubation and respiratory support may be needed if respiratory depression is present. No specific antidote to baclofen exists, however, if not contraindicated, physostigmine may be used to reduce central side effects such as somnolence and respiratory depression.[9] The treatment however, may not always be effective.[10] Atropine administered intravenously has also been shown to produce an increase in cardiac output, ventilation and temperature in a patient with baclofen overdose.[11] There is no role for extracorporeal removal. Hemodialysis is the modality of choice for baclofen intoxication in patients with impaired renal function.[12] Full supportive treatment results in good outcome, provided no hypoxic or ischemic insult has occurred before medical attention.
